# Nutrition education for cardiovascular disease prevention in individuals with spinal cord injuries: study protocol for a randomized controlled trial

**DOI:** 10.1186/s13063-017-2263-2

**Published:** 2017-12-04

**Authors:** Jesse A. Lieberman, Jacquelyn W. McClelland, David C. Goff, Elizabeth Racine, Michael F. Dulin, William A. Bauman, Janet Niemeier, Mark A. Hirsch, H. James Norton, Charity G. Moore

**Affiliations:** 1 Carolinas Rehabilitation, 1100 Blythe Boulevard, Charlotte, NC 28203 USA; 2 North Carolina State University, Campus Box 7606, Raleigh, NC 27695 USA; 3 National, Heart, Lung, and Blood Institute, 6701 Rockledge Drive, Suite 8030, Bethesda, MD 20892 USA; 4 University of North Carolina at Charlotte, 9201 University City Boulevard, Charlotte, NC 28223 USA; 5 James J. Peters VA Medical Center, 130 West Kingsbridge Road, Bronx, NY 10468 USA; 6 Carolinas Medical Center, 1000 Blythe Boulevard, Charlotte, NC 28203 USA; 7 Center for Outcomes Research and Evaluation, Carolinas HealthCare System, 1540 Garden Terrace Road, Charlotte, NC 28203 USA

**Keywords:** Spinal cord injury, Nutrition education, Cardiovascular disease prevention

## Abstract

**Background:**

Individuals with chronic spinal cord injuries (SCIs) have an increased prevalence of cardiovascular disease (CVD) and associated risk factors compared with age-matched control subjects. Exercise has been shown to improve selected CVD risk factors in individuals with SCI, but using nutrition education as an intervention has not been evaluated in this population. This paper describes our research plan for evaluating the effect of nutrition education on individuals with SCI. In the present study, called *Eat Smart, Live Better,* we are using a randomized controlled design to test an intervention adapted from an existing evidence-based program that showed a positive effect on nutrition knowledge and behavior of older adults from the general population. There will be an inpatient group (*n* = 100) and a community group (*n* = 100). The aims of our study are to compare the intervention and control groups for (1) changes in nutritional behavior, nutritional knowledge, and dietary quality by participants in the program; (2) levels of adiposity and metabolic CVD risk factors at 12-month follow-up; and (3) differential effects among individuals with SCI in the acute rehabilitation setting and those living in the community.

**Methods/design:**

This is a randomized controlled trial of nutrition education. The treatment groups receive six nutrition education sessions. The control groups receive the one “standard of care” nutrition lecture that is required by the Commission on Accreditation of Rehabilitation Facilities. Treatment groups include both an inpatient group, comprising patients who have been admitted to an acute rehabilitation facility because of their recent SCI, and an outpatient group, consisting of community-dwelling adults who are at least 1 year after their SCI. A total of 200 participants will be randomized 1:1 to the intervention or control group, stratified by location (acute rehabilitation facility or community dwelling).

**Discussion:**

To our knowledge, this will be the first reported study of nutrition education in individuals with SCI. The low cost and feasibility of the intervention, if shown to improve nutritional behavior, suggests that it could be implemented in rehabilitation facilities across the country. This has the potential of lowering the burden of CVD and CVD risk factors in this high-risk population.

**Trial registration:**

ClinicalTrials.gov, NCT02368405. Registered on February 10, 2015.

## Background

Improved management of acute and chronic medical issues in individuals with spinal cord injuries (SCIs) has increased the prevalence of Americans living with chronic SCI in the United States [1, 2]. Persons with SCI have decreased energy expenditure compared with able-bodied individuals, in part owing to decreased activity but predominantly as a result of reduced resting energy expenditure because of the body composition changes that occur after SCI [3–5]. There is a significant loss of skeletal muscle and an increase in fat mass below the level of injury [6–9], as well as a decrease in sympathetic nervous system activity [10]. Subsequently, obesity, and particularly central adiposity, is common among persons with chronic SCI and is more prevalent than in able-bodied persons, with the greatest increase in weight often occurring during the first year postinjury [11–14]. In a cross-sectional study, 41.9% of adults with chronic SCI were found to have sarcopenic obesity [15]. In another cross-sectional study, 29–34% of the whole and regional body fat mass was accounted for by dietary fat intake [16].

In addition to obesity, persons with chronic SCI, regardless of the level of lesion, have an increased prevalence of metabolic risk factors for cardiovascular disease (CVD), such as dyslipidemia [17–19], glucose intolerance or diabetes mellitus [18, 20–26], central obesity [27–29], and systemic inflammation [22, 30–32], compared with matched able-bodied individuals. As a result, CVD is more prevalent and occurs prematurely in individuals with chronic SCI compared with the general population [1, 33–37]. Studies in the SCI population have shown that physical activity can positively affect a number of these risk factors [38–40]. A comprehensive obesity intervention using physical activity, behavior change techniques, and nutrition education resulted in significant weight loss [41]. However, persons with SCI face many barriers to being physically active [42–44], and other studies involving a physical activity intervention for CVD risk factors and body composition have not been as conclusive [45, 46]. Those with SCI may also have other potential barriers to nutritional sufficiency [47]. They may also have trouble getting to a grocery store or other places where fruits and vegetables, whole grains, and lean meats can be purchased; as such, they often rely on convenience and fast foods over home-cooked meals [48].

In contrast to physical activity, the relationship between nutrition education alone and metabolic CVD risk factors in those with SCI is not known. Dietary changes may be a more feasible approach for CVD prevention in persons with SCI. Dietary patterns consisting of prudent foods, specifically whole-grain foods, fruits and vegetables, and low-fat dairy products, are associated with decreased obesity, dyslipidemia, diabetes, and CVD in the general population [49–56]. Dietary quality is also inversely related to CVD risk factors in the general population [57].

Despite this evidence, persons with SCI have poor dietary intake of healthy foods and poor diet quality [58–64]. A study of men and women with chronic SCI in the community found inadequate intake of fiber, vitamin D, calcium, and potassium [59]. A study of men with chronic paraplegia found that their diets included total fat and saturated fat intake above recommended levels, whereas their fruit, fiber, calcium, and dairy intakes were below recommended levels [60]. A study of individuals with chronic SCI assessed their dietary intake and adherence to the American Heart Association (AHA) recommended dietary guidelines, and the researchers found that these individuals had excessive fat intake and inadequate fiber intake [62]. Findings were similar when the recommended Dietary Reference Intakes, the acceptable macronutrient distribution range [58], and the National Cholesterol Education Program recommendations were used [63]. Another study compared dietary intake among individuals with chronic SCI with age, sex, and race-matched, able-bodied control subjects as well as with the recommendations from the 2010 Dietary Guidelines for Americans. Individuals with chronic SCI consumed significantly less whole grain, fruit, and low-fat dairy, with only 8%, 39%, and 22%, respectively, meeting guideline recommendations for each food group [64]. The authors of many of these studies have suggested that more nutrition education during acute rehabilitation may improve dietary intake after discharge, but this question remains unanswered [65].

Nutrition education and its effects on nutrition behavior, diet quality, and CVD risk reduction have not been studied in persons with SCI. In addition, the best environment to deliver nutrition education to individuals with SCI is not known. Patients with SCI receiving nutrition education during acute rehabilitation may have difficulty retaining and applying learned dietary concepts when they return home. Second, individuals with SCI in the community may have difficulty with the transportation and caregiver support needed to attend education classes. For these reasons, the authors chose to study nutrition education delivered to both persons in acute rehabilitation facilities and to persons with chronic SCI (>1 year) living in the community.

In this randomized controlled trial (RCT), we are examining the effectiveness of a nutrition education program compared with usual care in two SCI cohorts (acute and chronic). The primary desired outcome is an improvement in nutrition behavior. Secondary outcomes include (1) increase in nutrition knowledge; (2) improvement in dietary quality, defined as increased intake of whole-grain foods, fruits, vegetables, and decreased intake of fat, cholesterol, sugar, and sodium; (3) less weight gain and increases in waist circumference (WC) and possibly weight loss and decreases in WC; and (4) improvement in metabolic CVD risk factors, such as cholesterol, triglycerides (TG), fasting blood sugar, and high-sensitivity C-reactive protein (hs-CRP).

This paper describes the design of a nutrition education program for individuals with SCI and the research study in which we will assess the effectiveness of this intervention. Our nutrition education program is a modification of the Eat Smart, Stay Well program, which has been shown to improve nutrition knowledge and behavior in older adults in the general population [66]. This program has been adapted by its founder, Dr. Jacquelyn McClelland, to target individuals with SCI and is entitled Eat Smart, Live Better. The goal of this study will be accomplished by the completion of three primary aims: (1) to compare changes in nutrition behavior, nutrition knowledge, and dietary quality of participants in the Eat Smart, Live Better program with those of subjects receiving usual care; (2) to compare weight gain, as well as changes in WC, fasting lipid profile, glucose, and hs-CRP, at 12-month follow-up between participants in the treatment groups and control subjects; and (3) to compare the differences in the intervention effect between the acute rehabilitation setting and the community-based setting.

## Methods/design

### Design

**Fig. 1:**
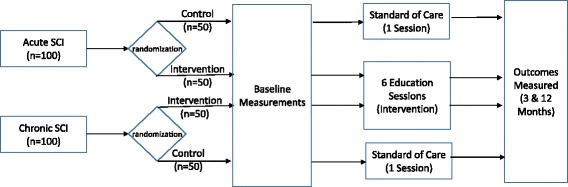
Research study design. The research study design is a randomized controlled trial. All measures are obtained at baseline (pretreatment) and repeated within approximately 3 months after their completion of their intervention (posttreatment). Additionally, measurements are repeated 12 months after the training intervention ceases (Outcomes Measured at 3 and 12 months). *SCI* Spinal cord injury

This study is an RCT with outcome assessor masking and repeated measures on the primary outcome (Fig. 1). Eat Smart, Live Well was chosen to be modified to Eat Smart, Live Better because of its effectiveness in improving nutrition behavior and knowledge and because its format and content are relatively easy to implement, so that, if successful, the program could be implemented in SCI rehabilitation centers across the United States.

### Setting

This study is being conducted at Carolinas Rehabilitation (CR; Charlotte, NC, USA), an acute inpatient rehabilitation hospital with a dedicated SCI rehabilitation unit and outpatient follow-up clinic. There are approximately 100 admissions for SCI each year. The most recent demographic analysis of the CR SCI Registry demonstrated a total of 287 patients with SCI, including 69.3% non-Hispanic white, 25.1% African American, 2.4% Hispanic, and 0.7% Asian patients.

### Participants and recruitment strategy

**Fig. 2:**
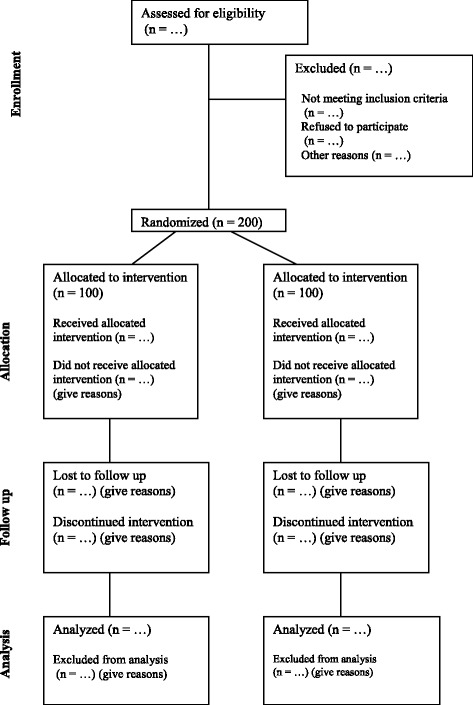
Consolidated Standards of Reporting Trials (CONSORT) flow diagram for the Nutrition Education for Cardiovascular Disease Prevention in Individuals with Spinal Cord Injury randomized controlled trial is shown, including the number of participants with acute and chronic spinal cord injury to be randomized

Participants will include men and women, regardless of ethnicity, ages 18–65 years. All of the acute participants will have American Spinal Injury Association (ASIA) classification A, B, or C as long as they have < 3/5 strength in up to three muscle groups, indicating that they will most likely not have functional ambulation [67]. Inclusion criteria for the participants with chronic SCI recruited from the community are ASIA C with no motor function limits if they do not functionally ambulate. Exclusion criteria are pregnancy (self-reported), end-stage renal disease, treatment for cancer except for nonmelanoma skin cancer within the past 5 years, and chronic nontobacco substance abuse. A total of 200 participants will be recruited and randomized. Of these, 100 participants (50 treatment, 50 control) will be in acute rehabilitation with an acute SCI, and 100 participants (50 treatment, 50 control) will be community-dwelling postacute individuals living with SCI (Fig. 2).

### Analysis

A medical history will be obtained from all participants, including level and completeness of SCI, previous treatment for CVD or other CVD, and other known CVD risk factors (e.g., hypertension, dyslipidemia, diabetes mellitus). They will also be queried concerning level of formal education, marital/partner status, and caregiver support for meal preparation.

### Recruitment strategy

In 2012, our rehabilitation hospital admitted 116 patients with acute traumatic SCI, 114 of whom presented with acute nontraumatic SCI. A review of the characteristics of this sample revealed that about 100 of these admissions would have met inclusion criteria for the proposed study. As a result, we expect to recruit a minimum of 25 inpatients per year in years 1–4. Community participants will be recruited through the CR SCI Registry. This registry was established for individuals with SCI who are interested in participating in research. We have been enrolling into our SCI registry over 50 individuals per year who are interested in participating in future studies, and we now have over 285 registered, of whom at least 125 are eligible for this study. We are continuously adding to this registry.

**Fig. 3:**
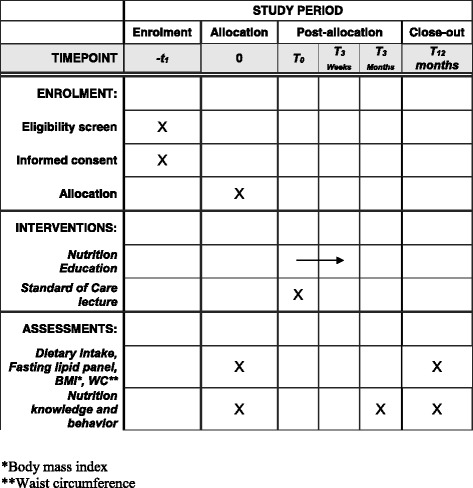
Standard Protocol Items: Recommendations for Interventional Trials (SPIRIT) figure

In order to thank participants for completing the study, each will receive $50 upon study completion. The Standard Protocol Items: Recommendations for Interventional Trials (SPIRIT) figure demonstrates the timing of the interventions and outcomes (Fig. 3).

### Intervention

The, Eat Smart, Stay Well program has traditionally been administered once per week for 5 weeks among older adults. Estimated delivery time for each session is 1 h. For the proposed study, Eat Smart, Stay Well has been modified to specifically target the SCI audience and has been retitled Eat Smart, Live Better. Intervention participants will participate in a series of six 60-minute small-group (one to four people, depending upon the SCI census) sessions of interactive nutrition education discussions led by a registered dietitian (RD) delivered over 3 weeks (average length of stay for persons with paraplegia). A sixth session was added as an introduction session because many items required by the Commission on Accreditation of Rehabilitation Facilities (CARF), such as what diabetes is and alcohol recommendations, need to be discussed with the inpatient group, as well as other aspects specific to SCI nutrition such as the need for fiber for bowel programs and fluid management.

### Intervention components

The interactive manualized curriculum follows the principles of the Dietary Approaches to Stop Hypertension (DASH) diet [68], MyPlate, the 2010 Dietary Guidelines for Americans [69] and the Nutrition Guidelines for Individuals with SCI [70]. The study program highlights the importance of eating a variety of foods from each food group and includes challenges for the participants to accomplish between sessions. The format for the intervention sessions includes weekly progress checkups, presentations/discussions, food preparation demonstrations, interactive hands-on skill-building activities and games, food taste testing, challenges, and peer group exchange. Small, frequent meals are emphasized to help with metabolism and to reduce volatility in blood glucose levels. All participants are encouraged to develop personal plans (goal setting) to make changes in dietary behaviors. The program design was based on the Health Belief Model [71], the Social Ecological Model [72], and the Theory of Planned Behavior [73]. The Health Belief Model purports that people choose to take or not to take preventive action depending on their perception of threats, benefits, and barriers. The program does not try to make participants fearful, but rather heightens their knowledge about health issues with which they already have concerns and discusses overcoming barriers and the benefits of taking action to decrease risks. We propose that the program’s use of discussions and interactive hands-on activities will be effective in increasing the needed knowledge and skills and decrease barriers, thereby increasing self-efficacy or confidence to make healthy behavior changes.

Knowing that participants’ perceptions/beliefs are not necessarily enough to move them to the action stage [71], Eat Smart, Stay Well addressed additional influences such as their economic limitations and relevant environmental factors, and these principles were adopted when designing Eat Smart, Live Better. Because an SCI can limit one’s earning ability, all of the recipes and suggestions are appropriate for someone with limited household funds. The Social Ecological Model takes into account the environmental factors that influence a person’s behaviors. The program is focused on individual (knowledge change, skill building, taste tests, increasing ability to overcome barriers) and interpersonal (peer-to-peer pressure and support) factors. Participants will be encouraged by the RD to contact each other and support each other. Caregivers and spouses of all of the participants are encouraged to attend the sessions. These theoretical models are based on the concepts that people are willing to make health-related behavior changes when they believe that their behaviors are placing them at risk of chronic disease (Health Belief Model); the environment plays a role in behavior change (Social Ecological Model); and by planning or making goals, people are more apt to accomplish the planned behavior (Theory of Planned Behavior).

The curriculum for Eat Smart, Live Better is generalized for anyone with an SCI. However, the program can also be customized. Each participant receives a study binder with the PowerPoint (Microsoft, Redmond, WA, USA) slides from each nutrition session. They also receive three sample daily food plans with a total daily caloric intake (*see* Appendix 1). The previously published estimated calorie-level calculations of 22.7 kcal/kg/day for those with tetraplegia and 27.9 kcal/kg/day for those with paraplegia published by Cox et al. will be used [74]. Ideal body weight will be calculated using the equation of Cox et al. (males, 5 pounds for the first 5 feet of height and 6 pounds for each further inch of height; females, 100 pounds for the first 5 feet of height and 5 pounds for each additional inch of height). These recommendations are meant only for those with acute SCI; however, there are not any other evidence-based calorie-calculating formulas.

It is emphasized that these are just recommendations and that the participants should not feel that they have to follow these advised levels of caloric intake strictly. This approach is being used because the goal of the program is for people to develop good nutrition habits that they will use for the rest of their lives, and not to feel that they are on a restricted diet. For those who have a pressure ulcer, the SCI evidence-based nutrition practice guideline recommends 30–40 kcal/kg/day [70].

For each food in the sample plan, the estimated dietary intake of protein, carbohydrate, fat, fiber, and calorie total is provided. These totals are also provided for each meal/snack to enable the participants to more easily understand how much of each macronutrient is being consumed. Each binder also includes many examples of breakfasts, lunches, dinners, and snacks along with the dietary intake information for each one. This is important because participants can mix and match meals/snacks and stay within their recommended calorie levels. The binder also includes worksheets from ChooseMyPlate.gov, including the MyPlate daily checklist for their designated calorie level. Other information in the binder includes tips for eating healthy at restaurants and cooking healthy at home, as well as information on when certain vegetables are in season.

### Nutrition sessions

#### Session 1: SCI, the basics

Participants are introduced to the CVD risk factors associated with SCI. The program explains why good nutrition is important for those with SCI for many reasons, including skin health, osteoporosis prevention, bowel management, and other issues. Participants are introduced to the MyPlate graphic and the food groups most emphasized in the program: whole grains, fruits, vegetables, low-fat dairy, and lean proteins. They are shown how each of these improves some individual CVD risk factors, such as central adiposity and blood sugar. The Nutrition Guidelines for Individuals with SCI are also introduced, and higher protein intakes are discussed in the context of eating balanced meals [70]. This is emphasized throughout the program. There is also a brief discussion of the benefits of physical activity.

#### Session 2: My eating plan

MyPlate and the food groups introduced in session 1 will be described in detail. Fats and oils and their pros and cons also will be discussed. In accordance with AHA recommendations as well as other evidence, participants are encouraged to get their dietary fat from foods that contain polyunsaturated fats and monounsaturated fats such as polyunsaturated vegetable oils, fish, avocado, and mixed nuts as opposed to animal fat sources such as red meats and cheeses with high levels of saturated fat [75–78]. Examples of healthy, convenient meals and snacks incorporating these foods will be introduced, and the sample meal plans in the study binder will be reviewed. Participants will make a toaster oven whole-grain pizza. They also will complete an action plan describing how they are going to improve their physical activity and their nutrition.

#### Session 3: Eating well challenges

This session is focused on overcoming barriers to healthy eating. Many common barriers to healthy eating, specifically to healthy eating with an SCI, are discussed along with ways to overcome them. Participants are encouraged to talk about barriers to healthy eating they have encountered in the past. Food package marketing strategies and some of the ploys that are used are also discussed. Participants learn how to make whole-grain, low-fat, low-sugar banana muffins. They are challenged to develop a strategy to overcome at least one barrier to healthy eating before the next session.

#### Session 4: Get what you need

In this session, participants will learn the importance of considering consumption of more of certain nutrients, such as calcium and vitamin D, because of low bone mineral density secondary to the unloading of the musculoskeletal system that occurs after SCI [79]. Protein in the context of building and maintaining lean mass is also discussed again. Participants will learn how to make a healthy tuna salad. They will be challenged to focus on eating enough of a variety of foods to ensure they are getting adequate amounts of the nutrients of interest before the next session. They will also be challenged to keep their caloric intake within their goal level.

#### Session 5: Choosing to win

In this session, reading and understanding food labels will be discussed. Participants also will be taught how to select health-enhancing foods when dining at restaurants, including fast food restaurants. They will learn how to make a tasty broccoli snack.

#### Session 6: Mapping out my destiny

The previous five sessions will be reviewed. Participants will develop their own personal nutrition plans to improve their health. They will be encouraged to use all of the concepts they have learned throughout the curriculum to complete this task. For their final recipe, they will learn how to make a corn and bean salad. The final challenge is for the participants to follow their personalized plan.

### Control intervention

The participants in the control group will receive the one standard nutrition lecture that is given to all patients with SCI during their acute inpatient rehabilitation stay at CR. CARF requires at least one session of nutrition education during acute SCI rehabilitation. This session will cover every nutrition-related topic required by CARF, including the increased risk of CVD and diabetes mellitus; the importance of nutrition for general health, skin integrity, and bowel management; and other basic nutrition advice. Once they have completed their 12-month data collection, participants in the control group will be encouraged to go through Eat Smart, Live Better. Participants from both groups will be informed that they should comply with their medication regimens and consume any food that they wish.

### Procedures for random assignment

After informed consent is obtained by the research assistant (RA), participants will be screened for eligibility. Because traumatic brain injury and SCI commonly occur together [80–83], participants must receive a Mini Mental Status Examination score > 23 in order to be eligible for study inclusion [84]. Eligible persons will be randomized to receive either the six Eat Smart, Live Better nutrition education classes or the standard nutrition education course currently required by CARF. The time schedule of enrollment in the interventions and outcome measures is demonstrated in Fig. 1.

Participants will be randomized to one of the two groups on the basis of computer-generated random numbers in SAS version 9.3 software (SAS Institute, Cary, NC, USA) by our biostatistician using permuted blocks. Assignments will be placed into an opaque envelope with the participant number on the outside by a volunteer not otherwise associated with the study. The participants will open this (or receive assistance if they are unable to) after they have completed their informed consent. Only the principal investigator (PI) and RD teaching the nutrition education courses will know to which group each participant has been assigned. To reduce bias, the RA collecting data will be masked. This may be difficult at times because there is only one paid RA, but a protocol will be developed to prevent unmasking. Should unmasking occur, a volunteer will administer the 3-month and 12-month nutrition knowledge and behavior questions. Success of the masking will be tested by querying the data collector about participant allocation.

### Outcomes

#### Nutrition behavior and knowledge (primary outcomes)

The following factors will be assessed at baseline, 3 months, and at 12 months. The Eat Smart, Stay Well program has an established set of 15 pre- and posttest questions for nutritional knowledge and behavior [66]. Nutritional behavior will be recorded from 15 five-point Likert scale questions ranging from 1 = not practicing the behavior to 5 = practicing it regularly. Questions will be related to eating fruits and vegetables and to decreasing fat intake. The recorded scores for these questions will be summed for nutritional behavior and range from 15 = very low to 75 = very high [66]. Nutritional knowledge will be assessed with knowledge-based questions with the answer choices of “true,” “false,” and “don’t know.” These instruments have been modified to include the changes that were made to the program to better assess the effects of nutrition education on nutritional behavior and knowledge.

#### Diet quality (secondary outcome)

The following factors will be assessed at baseline and at 12 months. To assess diet quality (excluding time spent in the hospital for the rehabilitation facility group), the Diet History Questionnaire (DHQ) II [85, 86] will be administered. Although the DHQ II has not been validated, the DHQ I has been validated extensively, and the changes made to generate the DHQ II were minimal. As such, the investigators are fairly confident that the validity of the measure has not been altered [86, 87]. Each participant will be assessed for fruit, vegetable, dairy, whole grain, lean protein, fat, and saturated fat intake. Intake for each food group will also be checked for compliance with the 2015 Dietary Guidelines for Americans.

#### Body mass index (secondary outcome)

The following factors will be assessed at baseline and at 12 months. Height and weight measurements will be obtained to calculate body mass index (BMI). Each participant will be weighed in their wheelchair using a commercial wheelchair calibrated scale, and the weight of the wheelchair will be subtracted. Height will be measured with the patient in supine position on a padded plinth using a flexible, nonelastic tape measure (Gulick II Tape Measure; Country Technology, Gays Mills, WI, USA). In the case of lower extremity contractures, participants will be asked their height.

#### Waist circumference (secondary outcome)

The following factors will be assessed at baseline and at 12 months. WC has been shown to be correlated with visceral adipose tissue in persons with SCI [28]. WC will be measured with the patient in supine position laterally at the point midway between the iliac crest and the lowest lateral portion of the rib cage and anteriorly at the point midway between the xiphoid process of the sternum and the umbilicus. A flexible, nonelastic tape measure will be used. WC will be measured in centimeters.

#### Laboratory studies (secondary outcome)

The following factors will be assessed at baseline and at 12 months. Each participant will have a fasting venipuncture in order to measure metabolic CVD risk factors, including a lipid panel, glucose, and hs-CRP. Total cholesterol and TG will be determined using an enzymatic colorimetric test and high-density lipoprotein using a homogeneous enzymatic colorimetric test (Beckman Coulter, Brea, CA, USA). For participants with TG < 400 mg/dl, low-density lipoprotein cholesterol values are determined using the Friedewald equation [88].

Because of the metabolic changes and changes in adiposity during the first year after injury, only nutrition behavior, knowledge, and diet quality will be used for comparison between treatment groups (acute treatment versus community treatment). In addition to the repeat baseline measures, each subject will be administered a Physical Activity Scale for Individuals with Physical Disabilities (PASIPD). The PASIPD is a 13-item scale that quantifies different levels of physical activity (leisure, household, and work) performed over the previous week. The measure has been shown to have validity in SCI [89]. This will be administered at baseline for the community group and at the 12-month assessment in both groups in order to eliminate the effect of physical activity as a confounding factor on CVD risk factors and measures of adiposity. All participants will be interviewed about their medical history, including their SCI, previous treatment for coronary heart disease or other CVD, and other known CVD risk factors (e.g., hypertension, dyslipidemia, and diabetes mellitus).

### Safety outcome

Participants will be monitored for chronic conditions associated with catabolic states (e.g., pressure ulcers, chronic urinary tract infections, chronic pulmonary infections) to ensure that there is not a disproportionate number in either group.

### Statistical analysis

Descriptive statistics, including means and SDs as well as counts and percentages, will be calculated. Baseline and demographic variables will be compared univariately between intervention and control groups stratified by acute rehabilitation and community. We will employ an intention-to-treat analysis stratified by location (acute rehabilitation versus community). The primary analysis will be done using a linear regression model. The primary outcome variable will be the 12-month nutritional behavior scores. For the primary analysis, the independent variable is the treatment (Eat Smart, Live Better versus control). Similar regression models will be used to analyze changes in other secondary outcomes measured on an interval scale, including nutritional knowledge and diet quality as measured in part by daily servings of fruit, vegetables, dairy, whole grains, lean protein, fat, saturated fat, BMI, WC, and laboratory values. If the data are not normally distributed, the Kruskal-Wallis test will be employed, stratified by acute/community group. For nominal data, such as accordance with the 2015 Dietary Guidelines, the chi-square test or Fisher’s exact test (if any cell expected values are < 5) will be employed. To address the possible effects of missing values, sensitivity analysis will be performed using multiple imputation techniques. SAS version 9.3 software will be used for all analyses. A two-tailed *p* value < 0.05 will be considered statistically significant.

### Sample size

The total sample size will be 200 participants, comprising 50 participants in the inpatient treatment group, 50 in the outpatient treatment group, and 50 in each of the control groups. We plan to enroll at least 50 subjects per year in years 1–4. The sample size is based on the concept of effect size (Cohen’s *d*), defined as the difference between the means divided by the SD. The SD of this measure in the SCI population is not known. To detect an effect size of 0.65 (in the medium range), with α = 0.05 and a power of 80%, 39 subjects are required in each group [90]. To conservatively allow for 20% loss to follow-up, 50 subjects will be recruited into each arm of the study.

### Data quality and monitoring

Quality control will include regular data verification and protocol compliance checks by our institutional compliance officer. All hard data will be organized into de-identified folders and stored in a locked file cabinet in a locked office. The RA will enter all data into Research Electronic Data Capture (REDCap). All study data will be collected and managed using REDCap electronic data capture tools hosted at Carolinas Healthcare System [91]. REDCap is a secure, web-based application designed to support data capture for research studies by providing (1) an intuitive interface for validated data entry, (2) audit trails for tracking data manipulation and export procedures, (3) automated export procedures for seamless data downloads to common statistical packages, and (4) procedures for importing data from external sources. Only the RA, PI, and biostatistical team will have access to this database. After every tenth subject, data entry will be verified by the PI. There will not be any interim analysis. The Office of Clinical and Translation Research at our institution has a team of auditors that conduct random audits to ensure study binder, consents, and collected data are in order and all institutional review board (IRB) paperwork is accurately documented and stored correctly. Comprehensive and system-driven quality assurance audits are performed on a quarterly basis, focusing on key aspects of study processes that demonstrate compliance with standards (signed informed consent on the chart, subject fulfillment of inclusion/exclusion criteria, adverse event reporting). A report of the audit will be sent to the PI. If systematic errors are identified, staff retraining will occur and/or changes to procedures made as appropriate. Continuous quality improvement methods, in conjunction with performance indicators, will be instituted to monitor day-to-day activities of our project.

The PI and the RA will be responsible for subject safety and data monitoring. The PI and RA will conduct and monitor the study in accordance with International Conference on Harmonization Good Clinical Practice Guideline. Responsibilities include study monitoring (review accuracy and completeness of records, source document checks), evaluation of study data (laboratory data, communication, and written records), and adverse event monitoring. Concerns that might dictate study modifications or termination include participant safety, data quality, integrity, recruitment, and performance. Participants will be monitored for adverse events. All adverse events will be recorded on adverse event reporting forms, including action taken. Events determined by the PI to be unanticipated problems involving risks to subjects or others (UPIRTSO) (caveat) will be reported by the PI to the IRB within 10 days as per standard policy guidelines. Adverse events that are determined by the PI not to be a UPIRTSO will be reported per IRB policy at the time of continuing review. Serious adverse events will be reported to the IRB promptly and in no case later than 2 business days, as stated by the IRB policy. Periodic summary reports will be presented to our institutional safety officer (Dr. Vu Nguyen). The study has no data monitoring committee. Neither the National Heart, Lung, and Blood Institute nor the IRB required one secondary to the study’s very low risk of harm to the participants.

## Discussion

The overall objective of this research study is to determine whether a manualized, standardized nutrition education program is effective in improving nutritional knowledge and behavior among individuals with SCI and to determine the optimal environment for this education. This study is innovative in that it will be the first, to our knowledge, to identify the relationship between nutrition education and nutritional behavior, nutritional knowledge, diet quality, and metabolic CVD risk factors in the SCI population. Our central hypothesis is that the participants in the treatment group will improve their nutritional knowledge and behavior compared with those who receive standard care. The nutrition education intervention is a relatively low-cost intervention, and if shown to be effective, it will have the potential to be rapidly implemented in rehabilitation centers across the United States as well as other countries. If the nutrition education program is able to positively affect the CVD risk factors, then future studies can follow participants for a longer period of time to see if the incidence of diabetes or CVD can be decreased as well.

This study is not without limitations. The intervention briefly addresses the benefits of physical activity, but it does not emphasize this issue throughout the program. Therefore, it is not known what effect a combined nutrition and physical activity education program delivered during acute rehabilitation could have on CVD risk factors in individuals with SCI. Physical activity is also measured from participant recall, which can be subject to error. Another limitation is the content of the nutrition education program. The nutrition intervention is based on MyPlate and the 2015 Dietary Guidelines for Americans, although higher protein intakes are recommended in the Nutrition Guidelines for Individuals with SCI [70]. It is possible that another diet previously shown to improve CVD risk, such as the Mediterranean diet [92–94] or the DASH diet [68, 95], could have a greater effect on CVD risk factors and, ultimately, on preventing CVD.

In summary, the EatSmart, Live Better curriculum will test the effectiveness of a nutritional intervention to promote cardiovascular health in the SCI population, a population that is appreciated to be at higher risk for CVD than the general population. In addition to testing overall effectiveness, the trial will provide insight into whether the intervention is equally effective across inpatient and outpatient settings, information that will help guide subsequent implementation and dissemination should the intervention prove successful.

## Trial status

Recruitment began in April 2015. Recruitment will continue until April 2019 or until there are 100 participants in each study group. So far, 78 participants have been recruited.

## Appendix

For each food in the sample plan the estimated dietary intake of protein, carbohydrate, fat, fiber, and calorie total is provided. These totals are also provided for each meal/snack to enable the participants to more easily understand how much of each macronutrient is being consumed. Each binder also includes many examples of breakfasts, lunches, dinners, and snacks along with the dietary intake information for each one. This is important because participants can mix and match meals/snacks and stay within their recommended calorie levels. The binder also includes worksheets from chooseMyPlate.gov including the MyPlate daily checklist for their designated calorie level. Other information in the binder includes tips for eating healthy at restaurants, cooking healthy at home, and information on when certain vegetables are in season.

## Abbreviations

AHA: American Heart Association
ASIA: American Spinal Injury Association
BMI: Body mass index
CARF: Commission on Accreditation of Rehabilitation Facilities
CONSORT: Consolidated Standards of Reporting Trials
CR: Carolinas Rehabilitation
CVD: Cardiovascular disease
DASH: Dietary Approaches to Stop Hypertension
DHQ: Diet History Questionnaire
hs-CRP: High-sensitivity C-reactive protein
IRB: Institutional review board
PASIPD: Physical Activity Scale for Individuals with Physical Disabilities
PI: Principal investigator
RA: Research assistant
RCT: Randomized controlled trial
RD: Registered dietitian
REDCap: Research Electronic Data Capture
SCI: Spinal cord injury
SPIRIT: Standard Protocol Items: Recommendations for Interventional Trials
TG: Triglycerides
UPIRTSO: Unanticipated problems involving risk to subjects or others
WC: Waist circumference
